# Supplementary material for: A quantitative binding model for the Apl protein, the dual purpose recombination-directionality factor and lysis-lysogeny regulator of bacteriophage 186
Source: Nucleic Acids Res. 2020 Aug 13;48(16):8914–26. doi: 10.1093/nar/gkaa655 (PMC7498355; doi:10.1093/nar/gkaa655)
Supplement: gkaa655_Supplemental_File [file gkaa655_supplemental_file.pdf]

SUPPLEMENTARY MATERIAL

1. Supplementary Figures

Oligonucleotides for Apl binding

A. pR-pL

Apl 2

TTGA**TGGCAA**GTGT**TGGCAA**ACAG

AACT**ACCGTT**CACA**ACCGTT**TGTC

Apl 3

TTGA**TGGCAA**GTGT**TGGCAA**ACAG**AGTCAA**ATCA

AACT**ACCGTT**CACA**ACCGTT**TGTC**TCAGTT**TAGT

Apl 4

TTGA**TGGCAA**GTGT**TGGCAA**ACAG**AGTCAA**ATCAA**TTGCAA**ACTT

AACT**ACCGTT**CACA**ACCGTT**TGTC**TCAGTT**TAGTT**AACGTT**TGAA

Apl 5

AACCC**ACGCAA**TTGA**TGGCAA**GTGT**TGGCAA**ACAG**AGTCAA**ATCAA**TTGCAA**ACTT

TTGGG**TGCGTT**AACT**ACCGTT**CACA**ACCGTT**TGTC**TCAGTT**TAGTT**AACGTT**TGAA

Apl 6

TATTT**TGGCTA**AACCC**ACGCAA**TTGA**TGGCAA**GTGT**TGGCAA**ACAG**AGTCAA**ATCAA**TTGCAA**ACTT

ATAAA**ACCGAT**TTGGG**TGCGTT**AACT**ACCGTT**CACA**ACCGTT**TGTC**TCAGTT**TAGTT**AACGTT**TGAA

Apl 7

TATTT**TGGCTA**AACCC**ACGCAA**TTGA**TGGCAA**GTGT**TGGCAA**ACAG**AGTCAA**ATCAA**TTGCAA**ACTT**TGGCTA**ATAGG

ATAAA**ACCGAT**TTGGG**TGCGTT**AACT**ACCGTT**CACA**ACCGTT**TGTC**TCAGTT**TAGTT**AACGTT**TGAA**ACCGAT**TATCC

Apl 101

TGA**TGGCAA**GTGT***CTTAGG***ACAG**AGTCAA**ATCA

ACT**ACCGTT**CACA***GAATCC***TGTC**TCAGTT**TAGT

Apl 1101

AACCC**ACGCAA**TTGA**TGGCAA**GTGT***CTTAGG***ACAG**AGTCAA**ATCA

TTGGG**TGCGTT**AACT**ACCGTT**CACA***GAATCC***TGTC**TCAGTT**TAGT

Apl 10101

AACCC**ACGCAA**TTGA***CTTAGG***GTGT**TGGCAA**ACAG***CTTAGG***ATCAA**TTGCAA**ACTT

TTGGG**TGCGTT**AACT***GAATCC***CACA**ACCGTT**TGTC***GAATCC***TAGTT**AACGTT**TGAA

Apl 1110111

TATTT**TGGCTA**AACCC**ACGCAA**TTGA**TGGCAA**GTGT***CTTAGG***ACAG**AGTCAA**ATCAA**TTGCAA**ACTT**TGGCTA**ATAGG

ATAAA**ACCGAT**TTGGG**TGCGTT**AACT**ACCGTT**CACA***GAATCC***TGTC**TCAGTT**TAGTT**AACGTT**TGAA**ACCGAT**TATCC

Apl 1101011

TATTT**TGGCTA**AACCC**ACGCAA**TTGA***CTTAGG***GTGT**TGGCAA**ACAG***CTTAGG***ATCAA**TTGCAA**ACTT**TGGCTA**ATAGG

ATAAA**ACCGAT**TTGGG**TGCGTT**AACT***GAATCC***CACA**ACCGTT**TGTC***GAATCC***TAGTT**AACGTT**TGAA**ACCGAT**TATCC

B. *att*P

*att*P 5

ATCA**TTGACA**TAGA**TTGCCA**TTGT**TTGCCA**ATAA**TTACCT**ATTA**TTCGCC**ATTTC

TAGT**AACTGT**ATCT**AACGGT**AACA**AACGGT**TATT**AATGGA**TAAT**AAGCGG**TAAAG

Supplementary Figure 1. Apl binding site sequences used to assess Apl binding at the pR-pL region (A) or *att*P region (B). Double stranded sequences containing Apl binding sites (bold) or scrambled sites (bold, italics) are shown. Underlined sequences indicate the oligonucleotides used to generate the double stranded sequences.

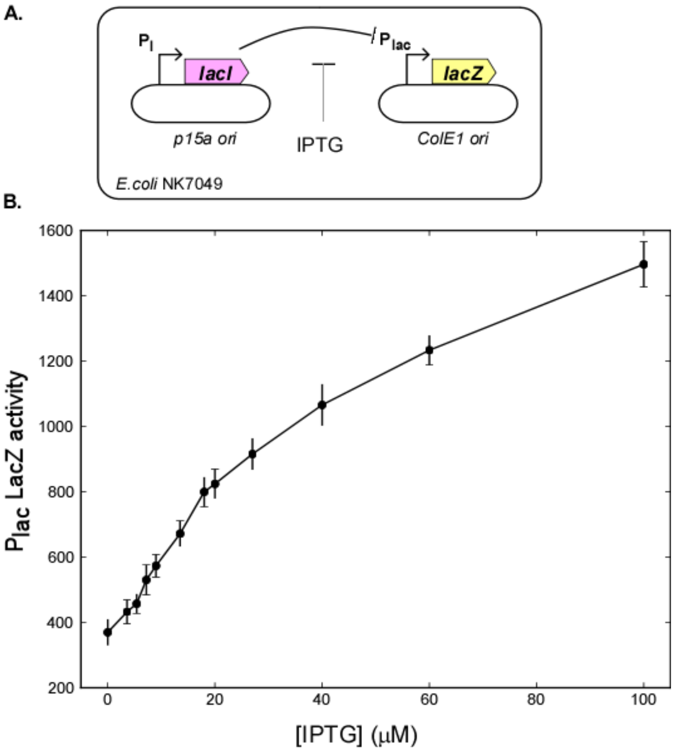

Supplementary Figure 2. Relationship between IPTG concentration and pLac activity used to estimate relative expression levels of Apl *in vivo*. The *in vivo* Apl expression system (Fig. 5) used an identical plasmid set up to that shown here, where the *lacZ* gene has replaced the *apl* gene (A). Thus, relative Apl expression units at different IPTG concentrations can be obtained by using the LacZ vs IPTG plot shown here (B). Error bars represent 95% confidence intervals.

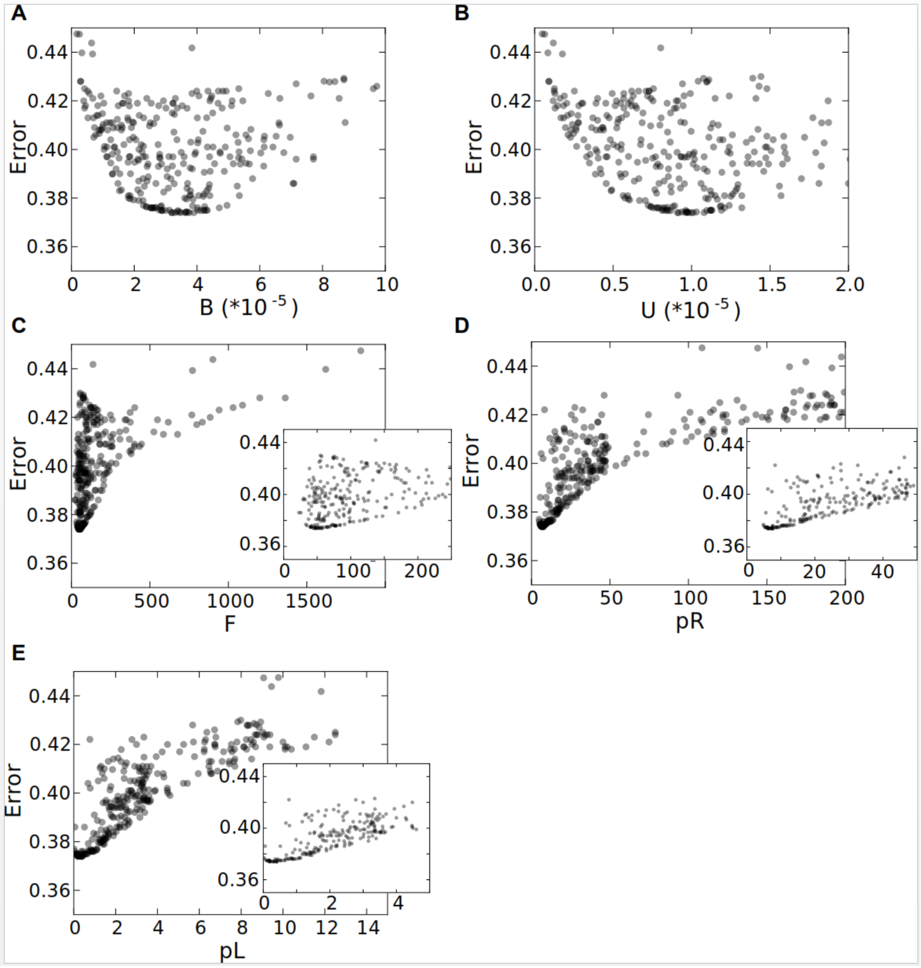

Supplementary Figure 3. Optimisation of best fit parameters.

Error terms in the fitting are plotted against the values of the parameters for specific Apl binding (panel A), non-specific binding (panel B), cooperativity (panel C), pR (panel D) and pL (panel E). The plots show that the fitting procedure converges to minimise the error term. Insets in panels C-E show more detail.

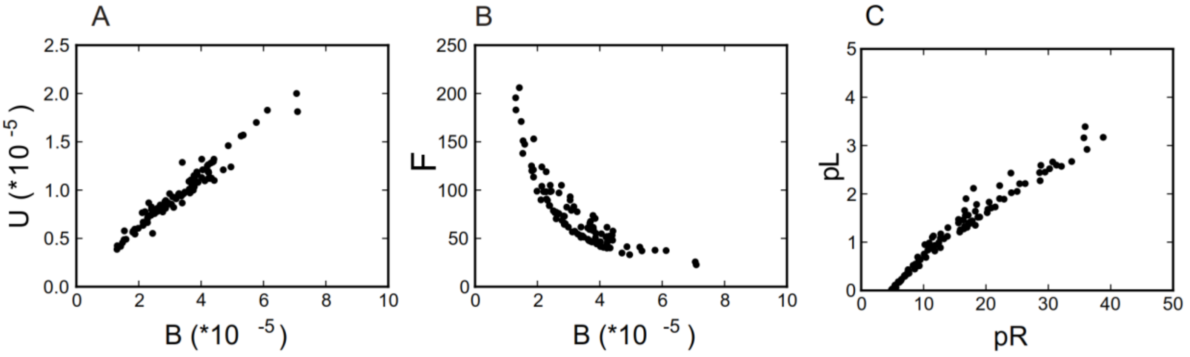

Supplementary Figure 4. Correlation between fitted parameters.

Parameter correlations for the best 100 fits of the model to the lacZ data of Figure 5 are shown here.
(Panel A) Non-specific binding (U) increases linearly with specific binding strength (B).
(Panel B) Cooperativity (F) decreases as specific binding strength (B) increases.
(Panel C) Promoter strengths for pR increased linearly with pL strength.

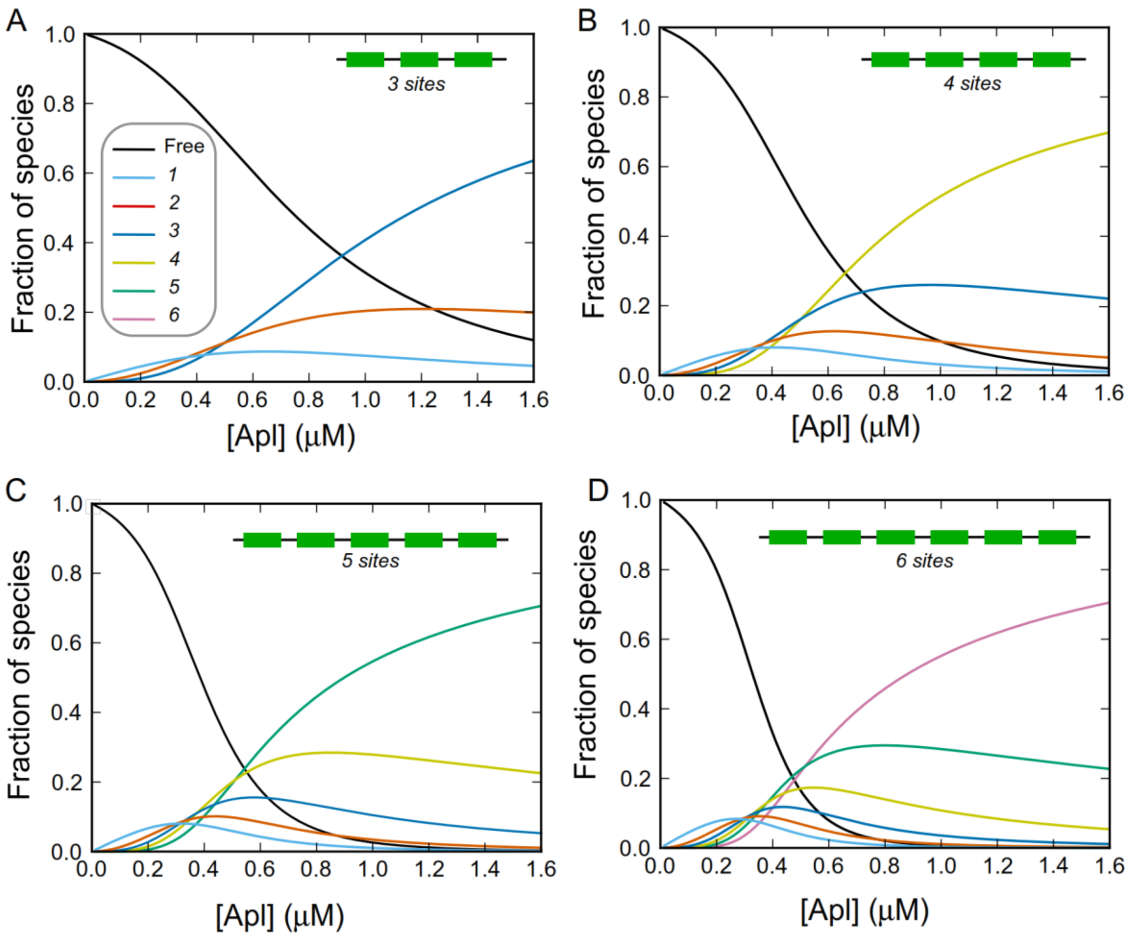

Supplementary Figure 5. Species plots for DNA containing three to six Apl binding sites.

The predicted distributions of binding stoichiometry as a function of Apl concentration are shown for DNA containing three (A), four (B), five (C) or six (D) Apl operators. The plots are colour coded according to the inset in panel A.

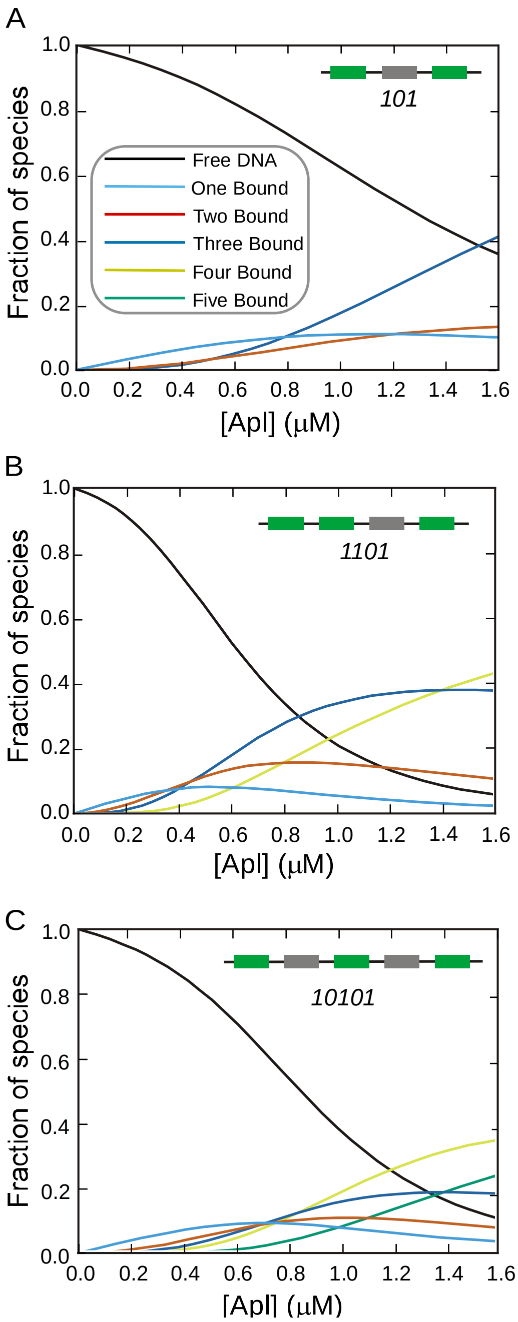

Supplementary Figure 6. Species plots for DNA containing non-specific sites.

Comparison of the predicted distributions of binding stoichiometry as a function of Apl concentration are shown for DNA containing three (A), four (B) or five (C) operators, with intervening scrambled operators (grey boxes).

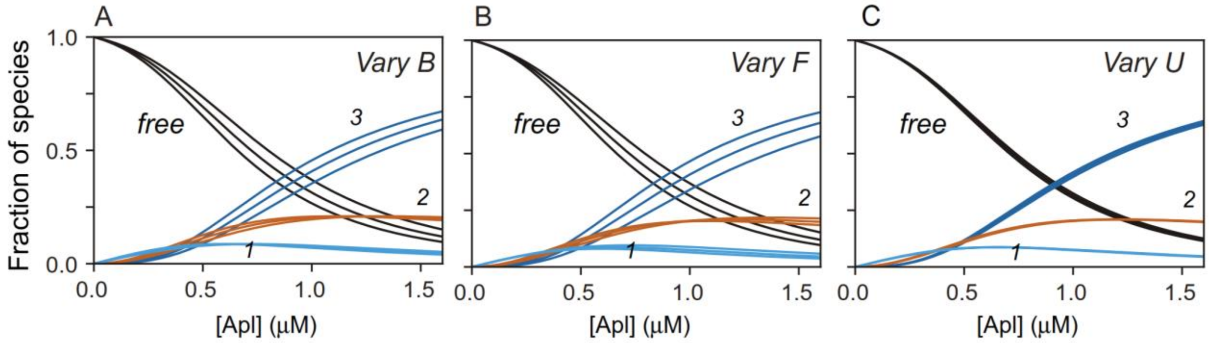

Supplementary Figure 7. Effect of parameter variation on species distributions.

The range of species distributions are plotted for the case of three consecutive Apl operators. For each plot, two of the three parameters are held fixed at their mean values and the third parameter varied higher and lower by one standard deviation. The standard deviation of the parameters was obtained from the best 100 fits to the *in vivo* data shown in Figure 5. (A) Fixed F, U; varied B. (B) Fixed B, U; varied F. (C) Fixed B, F; varied U. As expected, variation in U (panel C) has no impact when the DNA contains only specific sites.
